# Pilot Study of Chronic Obstructive Pulmonary Disease in an Industrial Town in India

**DOI:** 10.5696/2156-9614-9.21.190304

**Published:** 2019-03-07

**Authors:** Arun Kumar Sharma, Om Prakash Kalra, Narinder Kumar Saini, Harshita Kelkar

**Affiliations:** 1 Department of Community Medicine, University College of Medical Sciences and Guru Teg Bahadur Hospital, Delhi, India; 2 Pandit Bhagwat Dayal Sharma University of Health Sciences, Rohtak, India

**Keywords:** COPD, air pollution, spatial epidemiology, spirometry

## Abstract

**Background.:**

The burden of chronic obstructive pulmonary disease (COPD) in India is not well understood. Due to geographical and environmental heterogeneity, the epidemiological profile of COPD may not be uniform across the country. Studies carried out in small geographical areas can help to determine the prevalence and risk factors of COPD.

**Objectives.:**

The present study was conducted in one city in northwest India in order to calculate prevalence in small geographically determined areas within the city as well as across socio-economic strata and adjoining neighborhoods.

**Methods.:**

The present study was conducted in Ludhiana, an industrial town in Punjab, India. Residential colonies were identified in an industrial and nonindustrial area and all households were screened for COPD using the Global Initiative for Chronic Obstructive Lung Disease criteria and confirmed by spirometry. Information about exposure to possible risk factors was also collected from suspected cases of COPD. Cases were mapped on a digital map of the city and hotspots were identified.

**Results.:**

Fifty-six cases of COPD were detected. More than half (71%) were in the industrial area. The overall prevalence rate of COPD in the city was 3.17 per 1 000. The highest prevalence (5.6–9.4 per thousand) was observed in the colonies of the industrial area. All surveyed colonies in the nonindustrial area showed a low prevalence (0.0 to 0.9 per thousand).

**Conclusions.:**

Hotspots were located in the industrial area and there was no such aggregation in the nonindustrial area. This suggests a potential association of industrial air pollutants with COPD. A strength of the present study is that it provides important baseline data. However, the study was limited, as it did not show a temporal association of exposure to air pollution and smoking with COPD.

**Participant Consent.:**

Obtained

**Ethics Approval.:**

The study was approved by the Institutional Ethics Committee for Human Research of the University College of Medical Sciences, Delhi, India.

**Informed Consent.:**

Obtained

**Competing Interests.:**

The authors declare no competing financial interests.

## Introduction

Chronic obstructive pulmonary disease (COPD) is predicted to become the third leading cause of death by 2030.^1^ Low- and middle-income countries shoulder much of the burden of COPD and it is a major public health problem in India.^2^ However, there is no burden of disease estimation for COPD in India and the current prevalence pattern is not well understood.^3^ A literature search showed that studies are often based on hospital data or data collected from small geographical areas covering a very small population. Even district or state level estimations are absent from published literature. The estimated prevalence of COPD in India is between 6.5–7.7% as reported by McKay et al. in a systematic review.^3^ These studies are spread over 30 years and do not reflect the true epidemiological profile of COPD in India. This also points to the incompleteness of data as well as lack of representativeness in terms of geographical and environmental heterogeneity which significantly determine the distribution of COPD. Furthermore, given the geographical, socio-economic and environmental diversity, the epidemiological profile of COPD may not be uniform across the country, and therefore it may not be possible to have a one-size-fits-all approach to policy making and planning interventions for prevention, control and treatment of COPD. Several studies have concluded that COPD distribution greatly differs across geographical disparities.^4–6^ However, heterogeneity may not be solely geographical and could be due to underlying differences in socioeconomic status, racial and ethnic dissimilarities. A study by Chan et al. showed that in Taiwan, aborigines had a higher risk of COPD and therefore areas with a higher aboriginal population had more cases of COPD.^5^ Understanding geographic variations in COPD hospitalization could help public health policy makers more clearly identify target areas with greater needs and facilitate better solutions to improve COPD prevention and treatment strategies.^6^

Thus, in order to develop locally relevant information about the prevalence, risk factors and access to therapeutic services, studies are needed in smaller areas. As a first step, the present study was conducted in one city in northwest India in order to calculate prevalence in small geographically determined areas within the city as well as across socio-economic strata and adjoining neighborhoods.

## Methods

The present study was conducted in Ludhiana, an industrial town in the state of Punjab in northwest India, located at 30.9° N 75.85° E. According to the 2011 census, the population was 1,618,879. The town is an industrial hub populated by small-scale industrial units that produce machine parts, auto parts, household appliances, hosiery, apparel and garments. The two industrial areas are located in the eastern part of the city. Residential colonies of workers are scattered between industrial units. The western part of the city is comprised of residential neighborhoods, commercial hubs and Asia's largest agricultural university campus.

**Abbreviations**
*COPD*: Chronic obstructive pulmonary disease

Since the objective was to determine a distribution pattern among cases associated with geography and probability of exposure to risk factors, a random sampling technique was not adopted in this study. A prior walkthrough was performed in one of the industrial areas and in the nonindustrial residential areas in the western part of the city. All residential colonies were identified. All households were screened in each of the identified colonies using a structured questionnaire for identifying probable cases of COPD based on the Global Initiative for Chronic Obstructive Lung Disease criteria.^7^ Households were visited three times on separate days and were excluded if contact could not be made. Demographic information was collected from each household. All suspected cases of COPD were administered a detailed questionnaire (*Supplemental Material*) to collect information about exposure to possible risk factors for COPD prior to the onset of the disease. Spirometry was carried out using a portable handheld spirometer (Spiromin) on all suspected cases who could perform the test properly. Written informed consent was obtained from respondents in each household prior to data collection and consent was also obtained individually from each suspected case of COPD prior to filling out the questionnaire and conducting spirometry. If prescriptions and hospital discharge summaries of patients were available, they were examined to confirm the diagnosis of COPD. Geographic coordinates of each household were noted using a Trimble Juno SA handheld GPS device.

The study was approved by the Institutional Ethics Committee for Human Research of the college University College of Medical Sciences. Participants were assured confidentiality and privacy of all collected information. Personal identifiers were removed prior to data analysis. Patient information sheets detailing the objectives, methodology and risk involved in participation and contact information of the investigators were provided to the participants.

### Quality control

Field investigators participated in a training course on data collection. The project fellow was trained by the principal investigator in administering pulmonary function tests using Spiromin with 20 volunteers priors to field work. It was the responsibility of the project fellow to supervise data collection by being present on site during the entire period of data collection.

### Data analysis

A digital map of Ludhiana district (Open Series Maps, 1:50 000) in the form of shapefiles was obtained from the Survey of India and used for mapping. Map plotting and spatial analysis was carried out using ArcGIS 10.1. Descriptive tables were created using SPSS version 20.0 (IBM SPSS Inc., Chicago, USA).

## Results

In the industrial area of the city, 1,999 households spread over 30 colonies were screened covering a total population of 9,509. In the commercial and residential areas, 1,900 households were screened, for a total of 8,128 participants. The prevalence rate of COPD across population groups is given in Table 1. A total of 56 cases of COPD were detected during the survey. Of these cases, 71% were in the industrial area and 53% were male. Most of the female cases were housewives.

**Table 1:** Surveyed Residential Areas by Population

Residential colonies	Households	Population
**Industrial area total**	**1999**	**9509**
Arya colony	51	215
Bhagat Singh Nagar	83	381
**Lower income area**	442	2237
Geeta Nagar	224	1091
Jamalpur	420	2108
Moti Nagar	437	1999
Sainik colony	48	193
Urban Estate	294	1285
**Urban nonindustrial area total**	**1900**	**8128**
Hargobind Nagar	550	2289
Kichlu Nagar	549	927
Kidwai Nagar	801	4912
**Total**	**3 899**	**17637**

**Figure 1:**
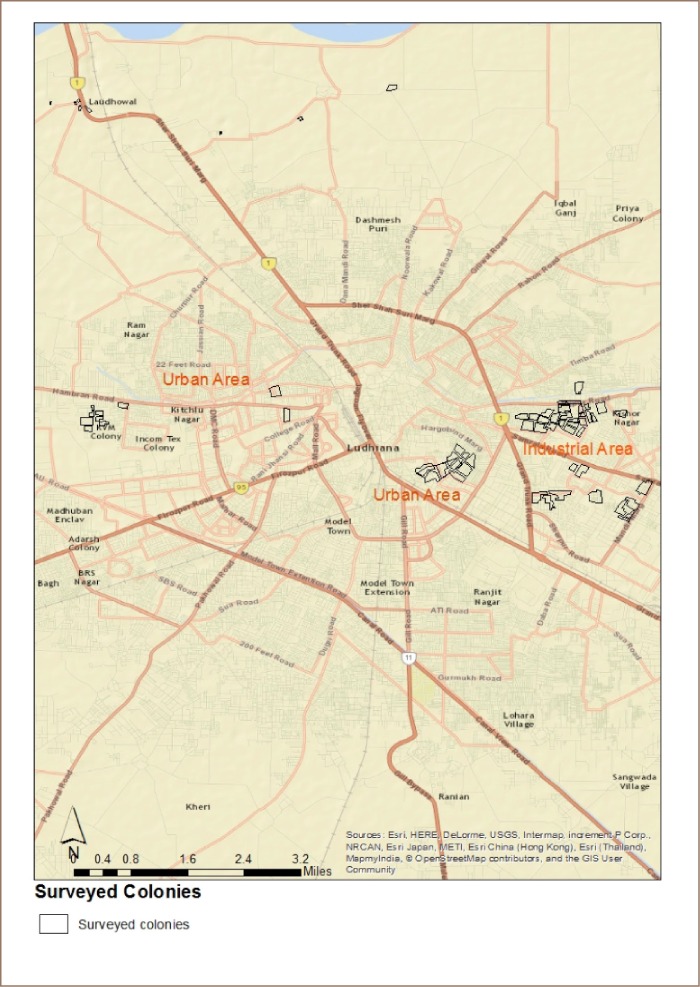
Colonies surveyed for chronic obstructive pulmonary disease in Ludhiana city

The surveyed colonies are shown on the map in Figure 1. In the industrial area, colonies were scattered between industrial units and included both small colonies, with only 35 households, and large colonies with 420 households. As the purpose was to identify COPD prevalence at the colony level, all households in selected colonies were screened for COPD. In the nonindustrial location, the response rate was very poor in middle- and high-income areas. In some colonies in the Civil Lines area and other affluent areas, the managers of the residential households did not give permission to conduct the survey and about 70% of the households in one wealthy area refused to participate. This was the most significant limitation of the present study.

**Figure 2:**
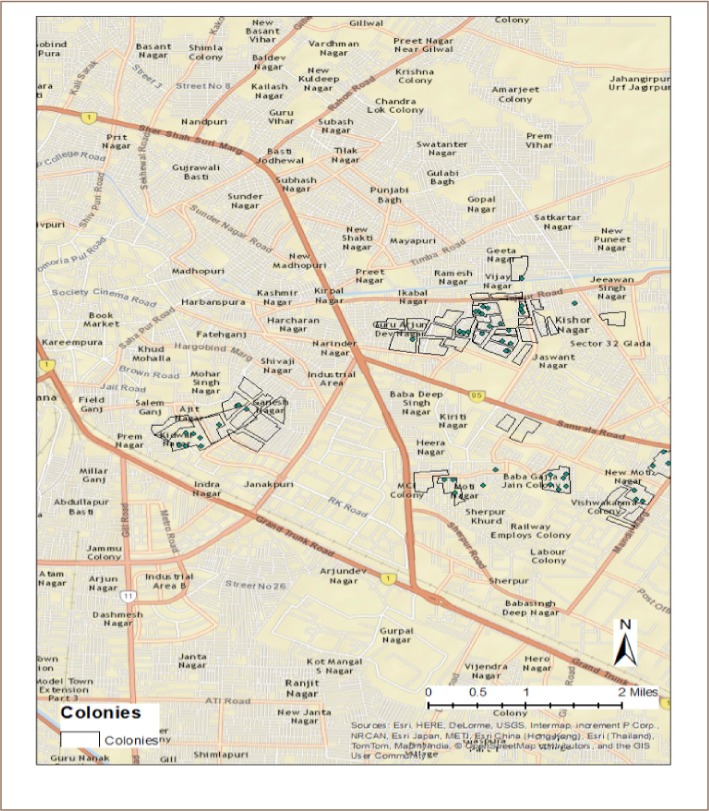
Map of Ludhiana showing surveyed areas and location of individual cases of chronic obstructive pulmonary disease (green dots)

**Figure 3:**
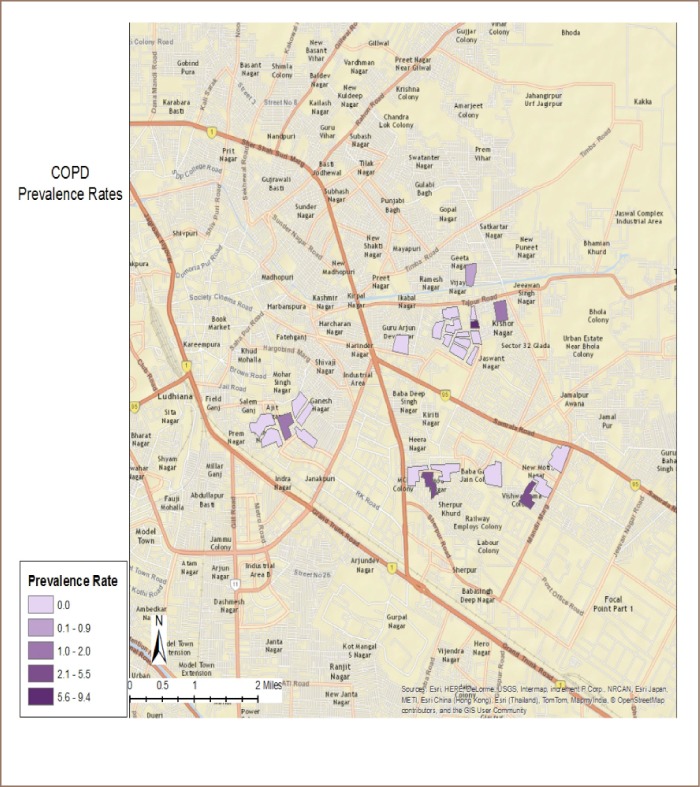
Colony-wide prevalence of chronic obstructive pulmonary disease cases in Ludhiana city

The location of individual cases is shown in Figure 2 where green dots represent the cases of COPD. The colony prevalence rate of COPD was calculated using spatial epidemiological techniques and raster-based modeling, as plotted in Figure 3. The highest COPD prevalence (5.6–9.4 per 1,000) was observed in the industrial area colonies. All surveyed colonies in the non-industrial area showed a low prevalence of COPD cases (0.0 to 0.9 per 1,000).

**Figure 4:**
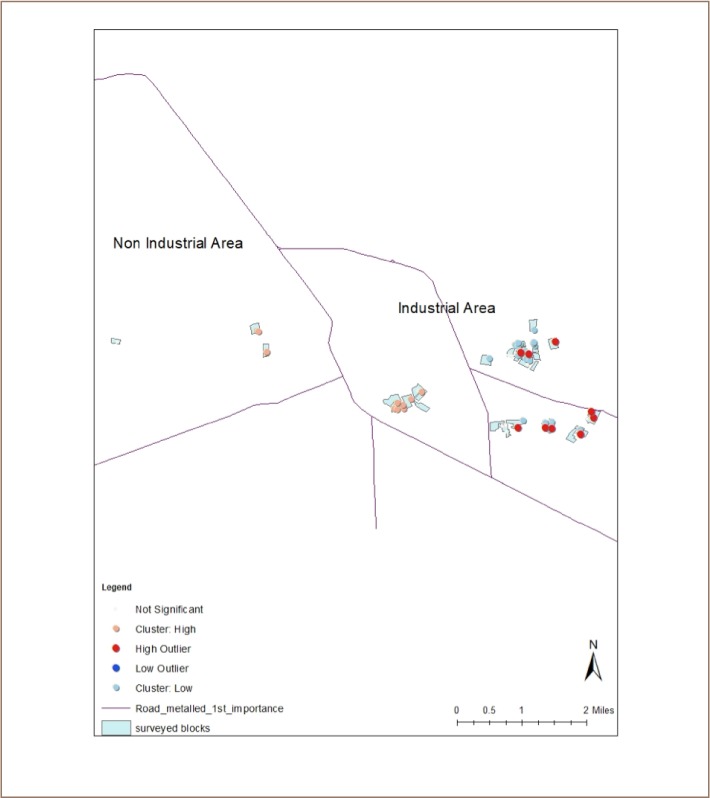
Hotspots of chronic obstructive pulmonary disease in Ludhiana city

Data in the present study help to identify the location of cases and determine hotspots. Hotspots are shown in Figure 4.

## Discussion

Ludhiana is one of the most polluted industrial towns in India. Due to the large number of manufacturing units located in Ludhiana, pollution levels are very high. The industrial area is interspersed with residential colonies, primarily composed of families who earn their livelihood from the nearby factories. The overall prevalence rate of COPD in the city of Ludhiana in our study was 3.17 per 1,000. To the best of our knowledge, there are no previously published studies of the prevalence of COPD in Ludhiana. McKay et al. stated in their review article that they had found no study from which an estimate of current prevalence of COPD in India could be drawn.^3^ Primarily due to differences in definitions used for diagnosis, age groups and screening methods across different studies (some studies used spirometry and others relied on questionnaire-based screening only), it is not possible to compare our findings with those of other studies.

The present study represents an initial attempt to determine the spatial determination of COPD in Ludhiana. Although the entire city was not covered, the surveyed areas are fairly representative of industrial areas and although incomplete, are nonetheless representative of nonindustrial areas as well. The visual distribution of cases gives an impression of a pattern, as the majority of cases are located in the industrial area of the city. Victor et al. reported greater than 20% variation in the prevalence and incidence of COPD from the provincial average in Ontario, Canada.^8^ In another study conducted in Ontario, Crighton et al. analyzed incidence and prevalence data for COPD from 2002 to 2011 and concluded that geographic heterogeneity exists at very small levels, with high prevalence in industrial areas within the city, namely Port Colborne and Hamilton.^4^ The spatial distribution pattern of COPD and asthma in England was described by Boulieri et al. and showed increasing effects from the south to the north of the country.^9^ The authors related this pattern with the prevalence of smoking in the northwest of the country and deprivation in the northeastern areas of the country. In that study, milder disease prevalence was associated with lower smoking rates and lower air pollution. This is similar to the finding in the present study of fewer cases in the urban/commercial part of the city compared to the industrial area. Lipton et al. reported geographical variability in COPD-related hospitalizations in California, with higher rates associated with areas of lower socio-economic status, poorer air quality and greater rurality.^10^

The prevalence of COPD also showed wide variation among colonies within the industrial and nonindustrial areas. This is an important observation for interventions to improve access to care for COPD patients as well as for the purpose of disseminating information to family members and neighbors. Hotspot analysis confirms this finding. The hotspots were located in the industrial area and there is no such aggregation in the nonindustrial area. Kauhl et al. emphasized the need for spatial scan statistics to identify future public health interventions.^11^ They also assessed COPD prevalence at the smallest spatial scale possible and reported strong local clustering. Niewiadomska et al. reported that in one region of Poland (Silesian Voivodeship), the incidence of COPD varied across districts.^12^

The most significant limitation of the present study was the nonindustrial area, in middle- and high-income groups, where the response rate was poor and permission was not given to conduct the survey. Smoking and ambient air pollution are major risk factors for COPD.^13^ Although longitudinal cohort studies are required to show the temporal association of exposures to air pollution and smoking with COPD, the present study did not have access to temporal data on air pollution in Ludhiana. For the purpose of establishing associations, longitudinal studies are required to examine levels of PM2.5, sulfur dioxide and nitrogen oxide with real time data along with incidence data of COPD. However, the present study was unable to gather such information.

## Conclusions

Hotspots of COPD prevalence were located in the industrial area of Ludhiana. This suggests that there is a potential association with industrial pollutants playing a role in the development of COPD. This study provides important baseline data, but one limitation of the present study is that it was unable to show a temporal association of exposure to air pollution and smoking with COPD.
