# Supplementary material for: Pilot Study of Chronic Obstructive Pulmonary Disease in an Industrial Town in India
Source: J Health Pollut. 2019 Mar 7;9(21):190304. doi: 10.5696/2156-9614-9.21.190304 (PMC6421949; doi:10.5696/2156-9614-9.21.190304)
Supplement: Supplementary file 1 [file hapn-9-21-190304_s01.docx]

**Supplemental Material**

**FIELD DATA COLLECTION FORM**

Individual respondent

1. ID No.
2. Household No.
3. Age
4. Sex
5. Exposure assessment
   1. Living in present house since_____months
   2. Living in this city since____months
   3. State of origin__________
   4. Time spent at home_____ per day (hours)
   5. Time spent at work place_____per day (hours)
   6. Time spent in commuting___per day (hours)
   7. Most often used modes of transport (Rank) (If respondent has COPD, then ask about mode of transport before diagnosis of illness)
      1. Walking
      2. Cycling/tri cycle rickshaw
      3. Auto
      4. Car/taxi
   8. Fuel used for cooking

LPG Kerosene Coal Wood Others

- - 1. Daily
    2. Less than daily
    3. Occasionally
    4. Never

1. Smoking status
   1. Do you currently smoke tobacco?
      1. Daily
      2. Less than daily
      3. Not at all
      4. No answer
   2. Have you smoked tobacco in the past?
      1. Daily
      2. Less than daily
      3. Not at all
      4. No answer
   3. Age at starting to smoke_______years
   4. Age at starting to smoke daily____
   5. Quantity of smoking (units/per day) (If not daily, write per week)
      1. Cigarette
      2. Bidi
      3. Hukkah (sessions)
      4. Others
   6. How long has it been since you stopped smoking?

________

- 1. Who are the other smokers in the family who are regularly staying with you?

List:

1.

2.

3.

4.

1. Industrial exposure
   1. What is your designation at work
   2. Does your work involve any of the following
      1. Exposure to pollutants
      2. Exposure to dust
      3. Exposure to heat
      4. Exposure to VOC
      5. Exposure to SPM
   3. How many hours in a normal working day are you exposed to above
      1. Exposure to pollutants
      2. Exposure to dust
      3. Exposure to heat
      4. Exposure to VOC
      5. Exposure to SPM
   4. How long have you been employed in this job?
   5. What was your designation prior to joining this job?
   6. How long did you hold that job?
   7. Did your previous job also involve such exposure?
   8. If yes, which of the following
      1. Exposure to pollutants
      2. Exposure to dust
      3. Exposure to heat
      4. Exposure to VOC
      5. Exposure to SPM
   9. How many hours in a normal working day are you exposed to above
      1. Exposure to pollutants
      2. Exposure to dust
      3. Exposure to heat
      4. Exposure to VOC
      5. Exposure to SPM

Screening for COPD

| Screening of COPD | Never  0 | Rarely  1 | Occasionally  2 | Often  3 | Frequently  4 | All the time  5 | Total |
| --- | --- | --- | --- | --- | --- | --- | --- |
| How often do you cough |  |  |  |  |  |  |  |
| Do you have mucus in your chest which comes out with coughing |  |  |  |  |  |  |  |
| How often do you feel tightness in chest? |  |  |  |  |  |  |  |
| How often do you feel breathless while climbing stairs? |  |  |  |  |  |  |  |
| Do you feel limited in doing activities of daily living due to lung conditions? |  |  |  |  |  |  |  |
| How difficult it is for you to move out of house due to your lung condition? |  |  |  |  |  |  |  |
| Do you have a sound sleep because of your lung condition? |  |  |  |  |  |  |  |
| Do you feel drained and weak due to your lung condition? |  |  |  |  |  |  |  |

Family history of COPD? Yes/No

If yes, list relationships:

Prescription/Diagnosis available: yes/no

If yes, source: Pvt. practitioner/hospital/ESI/Nursing home/RMP/others

Taking treatment: Yes/No

If no, why:

Willing to undergo Spirometry?

If no, why:

If yes: Spirometry done: Yes/No

If yes: report no

Interpretation of Spirometry

Do you suffer from any of the following illnesses? If yes, give the following details:

| Disease | Yes/No/ Don’t know | Since how many years | Taking treatment (Regularly/irregularly/ not started) | Remarks |
| --- | --- | --- | --- | --- |
| Diabetes mellitus |  |  |  |  |
| Hypertension |  |  |  |  |
| Coronary artery disease |  |  |  |  |
| Chronic kidney disease |  |  |  |  |
| Any mental illness |  |  |  |  |
